# Cropping practices, soil properties, pedotransfer functions and organic carbon storage at Kuanria canal command area in India

**DOI:** 10.1186/2193-1801-2-631

**Published:** 2013-11-23

**Authors:** Krishna Gopal Mandal, Dilip Kumar Kundu, Ravender Singh, Ashwani Kumar, Rajalaxmi Rout, Jyotiprakash Padhi, Pradipta Majhi, Dillip Kumar Sahoo

**Affiliations:** Directorate of Water Management (ICAR), Bhubaneswar, 751 023 Odisha India; Division of Crop Production, CRIJAF (ICAR), Barrackpore, 700 120 West Bengal India; Division of Agricultural Physics, IARI (ICAR), New Delhi, 110 012 India; Utkal University, Bhubaneswar, 751 007 Odisha India

**Keywords:** Cropping, Soil properties, Pedotransfer functions, SOC storage

## Abstract

Effects of cropping practices on soil properties viz. particle size distribution, pH, bulk density (BD), field capacity (FC, -33 kPa), permanent wilting point (PWP, -1500 kPa), available water capacity (AWC) and soil organic carbon (SOC) were assessed. The pedotransfer functions (PTFs) were developed for saturated hydraulic conductivity (Ks), water retention at FC and PWP of soils for different sites under major cropping system in a canal irrigated area. The results revealed that the soils are mainly composed of sand and clay with the clay contents ranging from 29.6 to 48.8%, BD of 1.44-1.72 Mg m^-3^, and 0.34 to 0.95% SOC. The Ks decreased, and water retention at FC, PWP and AWC increased significantly with soil depth due to greater clay contents in lower soil depths. The PTFs were best represented as the power functions for prediction of Ks with clay content as predictor variable; whereas the PTFs for water retention at FC and PWP were better represented as the exponential functions. SOC content was higher under rice-sugarcane crop rotation compared to other systems. SOC storage in the surface layer was higher in rice-sugarcane rotation (18.90-20.53 Mg ha^-1^) than other sites. The developed PTFs would be very useful in soil and water management strategies for the study area or elsewhere having similar soil and cropping practices. The information on SOC storage in the Kuanria region would help for better soil and crop planning in future.

## Introduction

The knowledge on soil physical properties is essential for land use planning, water resources management (Singh 2000; Kaur et al. 2001; Saikia and Singh 2003) and development of water harvesting structures in a canal irrigated command. Assessment of soil water regime is an important step in making water management decisions (Ungaro et al. 2005). Soil organic carbon (SOC) plays an important role for the functioning of agro-ecosystems. It influences the physical structure of the soil, the soil’s ability to store water, supply nutrients for crop production, and overall soil sustainability (Lal et al. 1997). The amount of SOC storage depends on soil texture, climate, vegetation, historical and current land use system (Mandal et al. 2012), and water regimes (Raut et al. 2012). This has more relevance in soils of the tropical and subtropical regions, including the Indian sub-continent (Bhattacharyya et al. 2000). Further, there is a need to understand the effects of irrigation on soil quality. There are different opinions by researchers on the effects of irrigation on soil quality. For example, Bendra et al. (2012) have found vulnerability on the changes in soil quality due to irrigation with greater volume to a light textured soil having poor organic matter content and slightly basic pH in northern America. On the contrary, Ricks Presley et al. (2004) noted several previous studies which concluded that irrigation caused no changes in soil physical properties but an equal number of studies that reported an impact of irrigation on soil physical properties. Reports are available that irrigation with sewage water improved the clay content, organic carbon and fertility status of soils (Friedel et al. 2000; Rattan et al. 2005). Singh et al. (2012) reported that domestic waste water with fertilizers has shown the improvement in the physicochemical properties of the soil, crop yield and also in the nutrient status in India. A decrease in hydraulic conductivity due to sewage irrigation has been reported by several researchers (Gonçalves et al. 2007; Masto et al. 2009).

The pedotransfer functions (PTFs) allow translation of textural information into estimation and/or prediction of hydrologic properties (Bouma and Finke 1993). As the direct measurement of hydraulic properties at multiple locations even within an agricultural field is time-consuming and costly (Romano and Palladino 2002), the indirect estimation of soil hydraulic properties and water retention using PTFs is very useful, as well as accurate and reliable (Ghanbarian and Millán 2010). Research on soil properties and development of models or PTFs has increased in recent years to improve the understanding of important soil processes, and evaluating the agricultural and environmental problems. Though, attempt to use PTF dates back to early years of the last century, Bouma (1989) called these functions as pedotransfer functions for the first time. Since then, the development of PTFs has been a continuous effort; results of such research have been reported (Liao et al. 2011; Abbasi et al. 2011). In India, researchers have developed the relationships or PTFs for field capacity (FC), permanent wilting point (PWP), saturated hydraulic conductivity (Ks) for some sites under Indo-Gangetic Plains (Bhavanarayana et al. 1986; Singh 2000; Kaur et al. 2001; Saikia and Singh 2003), and other soils (Adhikary et al. 2008; Chakraborty et al. 2011; Patil et al. 2012). These PTFs are not always applicable to every soil and crop production system. In eastern India, attempts have been made for the western catchment of the Chilika Lake, Odisha (Santra and Das 2008). But these studies have not addressed any specific area or a canal command where site-specific water resource management receives greater attention.

Furthermore, for a canal irrigated command like Kuanria Irrigation Project (KIP) as the study area, no attempt has been made so far for characterizing the soil properties, assessing soil organic carbon (SOC) storage and studies on PTFs. Cropping effects on soil properties and SOC storage in this command needs to be studied. The site-specific soil characterization and development of relationships are essential for proper land use planning and management of water. Therefore, the objectives of this study were: i) to assess the soil properties for different sites under major cropping systems being followed in the KIP command, ii) to assess the soil organic carbon and its distribution in soil profile, and iii) to develop site-specific pedotransfer functions to predict hydraulic conductivity and water retention characteristics of soils of Kuanria command area.

## Materials and methods

### The study site

**Figure 1:**
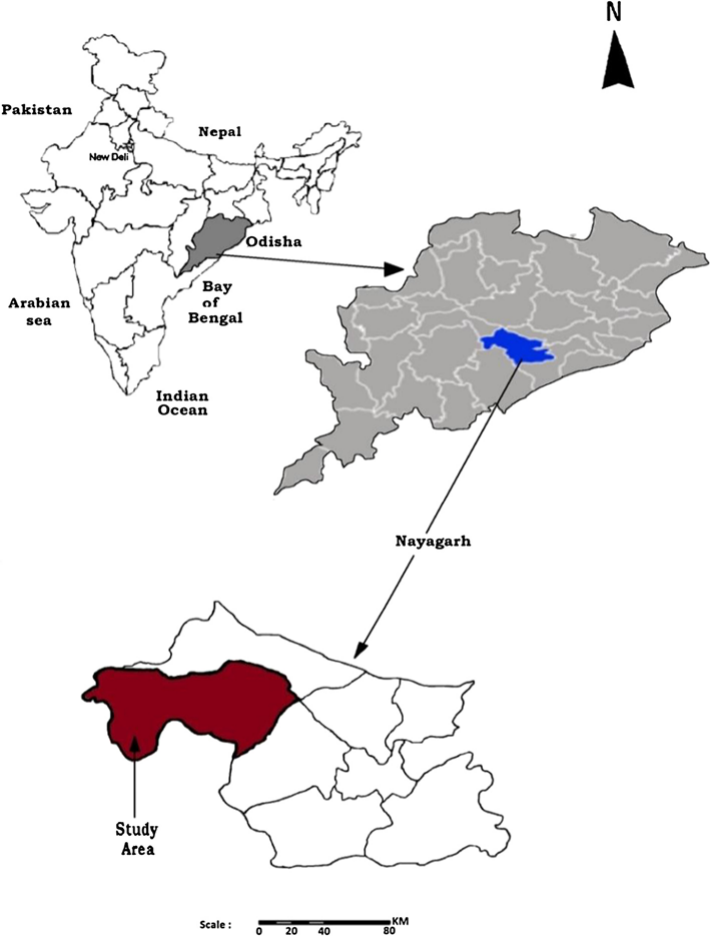
**The study area in Nayagarh district of Odisha, an eastern Indian state.**

The study was carried out on the canal commands of Kuanria Irrigation Project (KIP) at Nayagarh district of Odisha (Figure 1). Its main dam is located at 20° 21′ N latitude and 84° 51′ E longitude at an elevation of 122 m above mean sea level. The site belongs to Agro-Eco Sub-Region 12.2 (AESR 12.2) and Agro-Climatic Zone 7 (ACZ 7) of India according to NBSS&LUP (ICAR) and Planning Commission, Govt. of India classification, respectively. This area comes under a medium irrigation category in Odisha. This command area is intercepted by a river named ‘*Kuanria*’ which is a right tributary of a major river in India named ‘*Mahanadi’* and a ditch locally called as ‘*Khalakhala*’ form the reservoir of KIP. The storage capacity of the reservoir is 2200 ha m with a catchment area of 124 km^2^. The culturable command area is about four thousand ha. The command area covers Daspalla and Nuagaon blocks with geographical area of 571.57 and 385.24 km^2^, respectively of Nayagarh district in Odisha, an eastern Indian state.

### Climate and weather condition

The climate of the area is characterized by hot and moist sub-humid i.e., hot summers and cool winters. Total annual rainfall ranged from 993.5 to 1901.8 mm (average of 1995 to 2010 rainfall data) with an average of 1509.2 mm (CV 14.8%). Total annual effective rainfall was estimated as 858.2 mm which is 56.9% of the total annual rainfall. The normal southwest monsoon, which delivers about 75.7% of annual rainfall, extends from June to September of the year. This is also the main season (rainy season) for cultivation of rainfed crops; the other seasons viz. pre-monsoon (March-May), post-monsoon (October-December) and winter season (January-February) contributes only 10.8, 10.4 and 3.1% of the total annual rainfall, respectively. The temperature varies from a low of 10°C in winter to a high of 47°C during summer.

### Major cropping practices

The principal crop is rice, which is cultivated in the command in about 90.4% of the total area. Hence, the cropping system is predominantly rice-based. Rice is being grown during rainy season and green gram (10.6% of the total area) is mostly grown during post-monsoon season. Sugarcane is a major cash crop in the region. Pigeonpea is also grown in upland areas. Among vegetables, brinjal is leading; however, the cultivation of vegetables in the command is much less than rice. Rice, brinjal and mung bean (also known as green gram, *Vigna radiata* (L.) Wilczek, a pulse crop) occupy about 90.4, 8.8 and 10.6% of the total command area, respectively. The major cropping systems are presented in detail on varieties of crops and management practices (Table 1).

**Table 1:** **Major cropping systems - their location and crop management information**

Cropping systems, location	Cropping period	Crop variety, duration & spacing	Land preparation/tillage operations	Manure and fertilizer application	Irrigation	Average yield
Rice-fallow system (20° 18′ to 20° 21′ N, 84° 53′ to 84° 55′ E)	Rice (mid-Jun to end of Oct) in rainy season	Rice var. ‘Pratikshya (125 d) with 20 × 10 cm, ‘MTU 1001’ (120 d) with 20 × 10 cm	One summer ploughing and puddling before transplanting of rice	Farmyard manure (FYM) @ 3-5 t ha^-1^ yr^-1^, N-P_2_O_5_-K_2_O @ 60-30-30 kg ha^-1^	Supplemental irrigation 2-3 times during dry spells	2.8-3.2 t ha^-1^
Rice-sugarcane-2 years rotation (20° 20′ N, 84° 54′ E)	Rice (Jul-Nov) in rainy season; sugarcane (mid Apr- Feb/Mar)	Rice var. ‘Pratikshya (125 d), ‘Swarna’ (140-145 d), ‘MTU 1001’ (120 d) with 20 × 10 cm & sugarcane var. ‘Co 87044 (Uttara) Co 86249 (Bhavani) with 60-75 cm	One summer ploughing and puddling before transplanting of rice; ploughing and trenching while planting of sugarcane	FYM @ 3-5 t ha^-1^; N-P_2_O_5_-K_2_O @ 80-40-40 kg ha^-1^; FYM @ 5-7 t ha^-1^; N-P_2_O_5_-K_2_O @ 200-80-60 kg ha^-1^ in splits for sugarcane	Supplemental irrigation 4-5 times to rice; need periodical based irrigation to sugarcane	Rice yield 3-4.5 t ha^-1^ & sugarcane 80-100 t ha^-1^
Rice-mung bean system (20° 20′ N, 84°52′ E)	Rice (Jul-Oct/Nov) in rainy & green gram (second fortnight of Nov-early Feb)	Rice var. Pratikshya (125 d), ‘Swarna’ (140-145 d), ‘MTU 1001’ (120 d) with 20 × 10 cm and mung bean var. ‘Sujata’ (65-70 d), ‘Samrat’ (75-80 d) with broadcasting	One summer ploughing and puddling before transplanting of rice, one ploughing for mung bean	FYM @ 3-5 t ha^-1^ for rice; N-P_2_O_5_-K_2_O @ 60-30-30 kg ha^-1^ for rice and 20-40-20 kg ha^-1^ for mung bean	Supplemental irrigation 3-4 times to rice and residual moisture or one irrigation to mung bean	Rice yield 3.0-3.5 t ha^-1^ & mung bean 0.5-0.7 t ha^-1^

### Soil sampling and methods of analyses

Soil samples were collected from two sites each of rice-fallow, rice-sugarcane and rice-mung bean cropping system. The exact location of different plots with respect to their latitude and longitude were measured with a GPS meter (model, Garmin eTrex Vista, Germany). Soil samples were collected during dry periods of the year 2010-11 and 2011-12. Samples were collected with the help of auger and down to the profile depth up to 90 cm from different locations in a zig-zag pattern and also from four depth increments (i.e. 0-15, 15-30, 30-60 and 60-90 cm). After collection, soil samples were processed properly for laboratory analyses. Soil particle size distribution was determined by the Bouyoucous hydrometer method and soil texture class was determined by following the procedure of USDA classification. Soil pH was measured with the help of a digital pH meter (pHTestr30, Malaysia). Soil moisture retention at field capacity (FC, -33 kPa) and permanent wilting point (PWP, -1500 kPa) were determined by a pressure plate apparatus (Eijkelkamp, Model 505). The available water capacity (AWC, cm^3^ cm^-3^) of soils, expressed as volume of water per unit volume of soil, was estimated as the difference between FC and PWP. Saturated hydraulic conductivity (Ks) was measured by constant head method. Five replicates of bulk density (BD, expressed as Mg m^-3^) samples down to the profile depth up to 90 cm were also collected using soil cores and core samplers (Eijkelkemp Agrisearch Equipment) from four different layers, though it was difficult in few locations. We scrapped and removed carefully the upper ~30 cm soil after taking its samples in some locations. Then core sampler was operated for access to 90- cm soil depths. Organic carbon was determined by wet digestion method (Walkley and Black 1934). SOC storage was calculated by using soil organic carbon content (SOC), bulk density (BD), and thickness of soil layer.

### Statistical analyses

The analysis of variance (ANOVA) technique was carried out on the data for each parameter as applicable to completely randomized design (Gomez and Gomez 1984). The significance of the treatment effect was determined using F-test at 5% level. The mean differences were compared using Duncan’s multiple range test (DMRT) at 5% level of probability. The pedotransfer functions were developed using standard methods. The information on the accuracy and performance of the developed pedotransfer functions (PTFs) were indicated with statistical parameters viz. the coefficient of determination i.e., the R^2^ value which measures the contribution of the function with independent variable (clay content) to the variation in response variable i.e., Ks, FC and PWP in our study; root mean square error (RMSE) to indicate the accuracy of the PTFs, and mean error (ME) to indicate the bias in the model (Chakraborty et al. 2011; McBratney et al. 2011). RMSE was calculated from the average square difference between the predicted value (*Ypred*) and the observed value (*Yobs*), i.e.,

and the mean error (ME) was the mean difference between the predicted and observed values, i.e.,

The values of R^2^ were found significant at 5% level; RMSE values were less indicating greater accuracy of PTFs; ME values were very close to zero indicating the least bias in the models.

## Results

### Soil properties, water retention and available water capacity in different cropping

Particle size fractions of soil i.e. the sand, silt and clay differed in different sites, and in different soil depths (Table 2). The soils are mainly composed of sand and clay; textural class was classified as sandy clay loam in the surface layer, clayey or sandy clay in the lower layers except that in site 4, where surface soils are clay loam. In general, sand contents ranged from 34.0 to 56.2%, clay from 29.6 to 48.8% depending on the soil depths and cropping systems. Depth-wise distribution showed a significantly decreasing trend in sand content towards lower soil layers in every cropping system; whereas clay contents increased gradually towards lower soil depths. Clay fractions were comparatively higher in the study sites 3 and 4 under rice-sugarcane crop rotations than those in other sites. Silt contents in these soils were low. The pH of the soil varied from 6.5 to 7.5. The surface soils of sites 1, 2 and 5 were slightly acidic (<7.0). In the study sites 2, 3 and 6 under rice-sugarcane crop rotation and rice-mung bean system, soil pH was above 7.0 in the depths up to 90 cm. With the increase in soil depth pH increased and tended to be in the range of neutral or slightly alkaline.

**Table 2:** **Particle size fractions i.e. sand, silt and clay contents of the profile soil and textural classes under different sites and major cropping systems in the command**

Site/cropping system	Soil depth (cm)	Particle size distribution			Textural class
		Sand (%)	Silt (%)	Clay (%)	
Site-1 (Rice-fallow cropping)	0-15	50.1a	15.9a	34.0c	scl
	15-30	42.7b	16.1a	41.2b	c
	30-60	39.0b	12.8a	48.2a	c
	60-90	38.7b	12.5a	48.8a	c
Site-2 (Rice-fallow cropping)	0-15	51.9a	13.5ab	34.6d	scl
	15-30	48.4b	13.9ab	37.7c	sl
	30-60	45.7c	14.5a	39.8b	sc
	60-90	44.8c	10.4b	44.8a	c
Site-3 (Rice-sugarcane crop rotation)	0-15	50.5a	15.9a	33.6c	scl
	15-30	43.3b	15.9a	40.7b	c
	30-60	38.8b	13.6ab	47.6a	c
	60-90	38.3b	11.0b	50.7a	c
Site-4 (Rice-sugarcane crop rotation)	0-15	42.6a	18.4a	39.0c	cl
	15-30	38.9b	15.7ab	45.4b	c
	30-60	37.4bc	13.8ab	48.8b	c
	60-90	34.0c	12.7b	53.3a	c
Site-5 (Rice- mung bean cropping)	0-15	54.6a	15.8a	29.6c	scl
	15-30	48.6a	14.6a	36.8b	sc
	30-60	43.3ab	13.1a	43.6a	c
	60-90	41.8bc	13.1a	45.2a	c
Site-6 (Rice- mung bean cropping)	0-15	56.2a	12.2a	31.6d	scl
	15-30	50.8b	14.7a	34.5c	scl
	30-60	48.5bc	14.0a	37.5b	sc
	60-90	46.6c	11.3a	42.2a	sc

Mean values with the same letter within a column under any site are not significantly different according to DMRT at P < 0.05.

**Figure 2:**
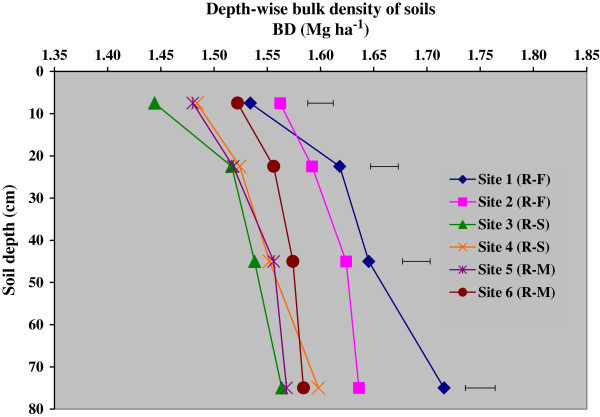
**Bulk density of soils in depth increments under different sites; R-F indicates rice-fallow, R-S rice-sugarcane rotation and R-M is rice-mung bean cropping; horizontal bars indicate LSD at 5% level of probability.**

The bulk density (BD) of soils in different soil depth was close to or greater than 1.5 Mg m^-3^ and it ranged from 1.44 to 1.72 Mg m^-3^, irrespective of different sites under major rice-based cropping in the command and soil depths (Figure 2). There was a slight difference in the BD in different cropping systems and sites in the upper soil layers. It indicated a comparatively lower BD in the sites 3, 4 with rice-sugarcane rotation and rice-mung bean cropping systems in site 5 relative to other sites and systems. For every site, BD increased with soil depth; however, this increase was higher in rice-fallow system in site 1. Overall, the soils of rice-fallow systems in sites 1 and 2 showed greater BD in every soil depth than other systems and study sites. The saturated hydraulic conductivity (Ks) of soils (Table 3) decreased significantly towards greater depths in the soil profile for each site under different cropping systems. This decrease was related to the greater clay contents in lower depths of soils. Among sites, Ks values were lower in the sites 3 and 4 where rice-sugarcane crop rotation is a prevalent system when compared to other sites. Evidently, higher clay contents were also observed in sites 3 and 4.

**Table 3:** **Saturated hydraulic conductivity (Ks) of soils in different sites under major cropping systems in the command area**

Soil depth (cm)	Saturated hydraulic conductivity (Ks) (cm h^-1^)					
	Site-1 (Rice-fallow cropping)	Site-2 (Rice-fallow cropping)	Site-3 (Rice-sugarcane crop rotation)	Site-4 (Rice-sugarcane crop rotation)	Site-5 (Rice-mung bean cropping)	Site-6 (Rice-mung bean cropping)
0-15	0.22a	0.22a	0.21a	0.17a	0.27a	0.24a
15-30	0.14b	0.17b	0.15b	0.14b	0.18b	0.21b
30-60	0.11b	0.15c	0.12b	0.12bc	0.13c	0.17c
60-90	0.11b	0.13d	0.11b	0.11c	0.12c	0.13d

Mean values with the same letter within a column under a site are not significantly different according to DMRT at P < 0.05.

**Figure 3:**
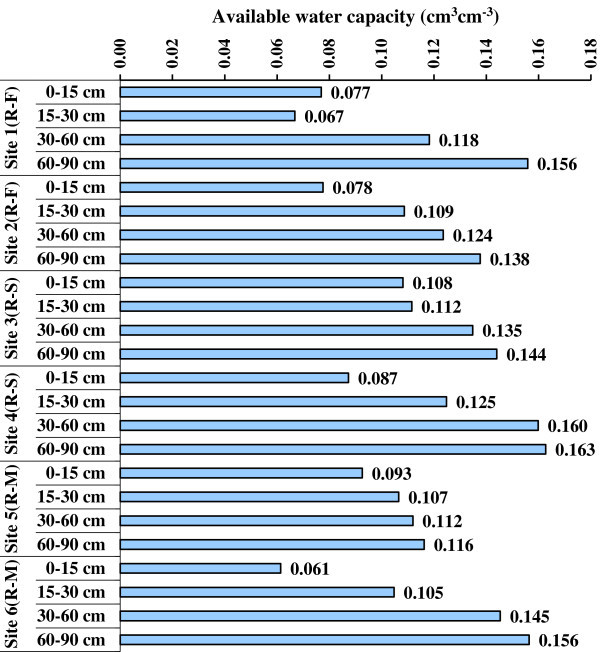
**Available water capacity (AWC) of soils in depth increments under different sites; R-F indicates rice-fallow system, R-S rice-sugarcane rotation and R-M is rice-mung bean cropping.**

The water retention at field capacity (FC, -33 kPa) and permanent wilting point (PWP, -1500 kPa) was slightly higher in sites 3 and 4 under rice-sugarcane crop rotation compared to other sites and cropping systems (Table 4). In general, FC ranged from 0.228 to 0.465 cm^3^ cm^-3^ irrespective of depth and site. Depth-wise water retention at FC showed a significantly greater retention with the increase in soil depth; and this trend was similar in every site and cropping system. The range of PWP was 0.161-0.308, 0.158-0.272, 0.222-0.279, 0.198-0.302, 0.163-0.254 and 0.169-0.236 cm^3^ cm^-3^ in sites 1 through 6, respectively (Table 4). Like FC, PWP increased significantly with the increase in soil depth for every site. The average PWP were greater in sites 3 and 4 under rice-sugarcane crop rotation that other sites. The AWC also was slightly greater in the soils of sites 3, 4 in the rice-sugarcane system than other systems; it showed higher values in the deeper soil layers (Figure 3). The difference in AWC was governed by the difference in FC and PWP; and basically, it is the clay fraction in the soils which determined the higher FC, and in turn the higher AWC of soils.

**Table 4:** **Field capacity and permanent wilting point of soils in different depths at different sites/cropping systems under the Kuanria command area**

Soil depth (cm)	Site-1 (Rice-fallow cropping)	Site-2 (Rice-fallow cropping)	Site-3 (Rice-sugarcane crop rotation)	Site-4 (Rice-sugarcane crop rotation)	Site-5 (Rice-mung bean cropping)	Site-6 (Rice-mung bean cropping)
*Field capacity (at -33 kPa) (cm* ^*3*^ *cm* ^*-3*^ *)*						
0-15	0.228b	0.236c	0.331c	0.286c	0.255d	0.231c
15-30	0.278b	0.348b	0.368b	0.370b	0.300c	0.297b
30-60	0.403a	0.393ab	0.406a	0.449a	0.339b	0.374a
60-90	0.464a	0.409a	0.423a	0.465a	0.371a	0.392a
*Permanent wilting point (-1500 kPa) (cm* ^*3*^ *cm* ^*-3*^ *)*						
0-15	0.161c	0.158c	0.222d	0.198c	0.163d	0.169c
15-30	0.211b	0.239b	0.257c	0.245b	0.194c	0.192b
30-60	0.285a	0.270a	0.272b	0.289a	0.227b	0.229a
60-90	0.308a	0.272a	0.279a	0.302a	0.254a	0.236a

Mean values with the same letter within a column under any study site are not significantly different according to DMRT at P < 0.05; a>b>c.

### Depth-wise distribution of soil organic carbon (SOC) storage

**Figure 4:**
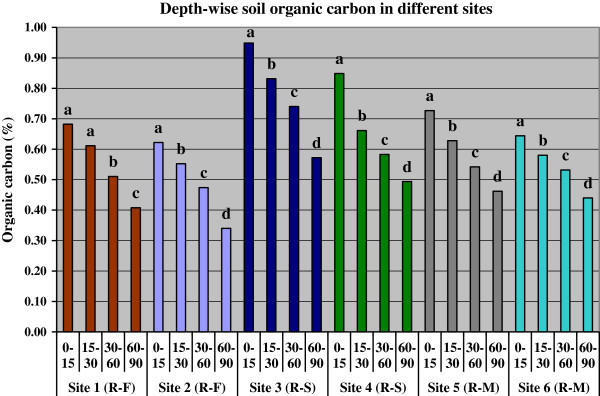
**Organic carbon content of soils in different depths under different sites; R-F indicates rice-fallow, R-S rice-sugarcane rotation and R-M is rice-mung bean cropping; vertical bars with same letter are not significant at p < 0.05 at a site as per DMRT.**

Soil organic carbon (SOC) content varied from 0.34 to 0.95% depending upon the soil layer and cropping systems (Figure 4). The SOC was highest in surface (0-15 cm) layer and then decreased down to the soil profile in every site. There was no sharp difference in SOC due to difference in cropping systems for every soil depths. However, one trend was clearly emerged out of the results that is, soils of site 3 and 4 under rice-sugarcane crop rotation had greater SOC content than other rice-based systems. In the surface soil (0-15 cm), SOC storage was higher in rice-sugarcane crop rotation systems; the values were 18.90 and 20.53 Mg ha^-1^ in the sites 3 and 4, respectively. However, other sites also had the organic carbon storage which amounts to 14.68, 16.16, 14.58 and 15.70 Mg ha^-1^ in the site 1 (rice-fallow) and site 2 (rice-fallow), site 5 (rice-mung bean) and site 6 (rice-mung bean), respectively.

**Figure 5:**
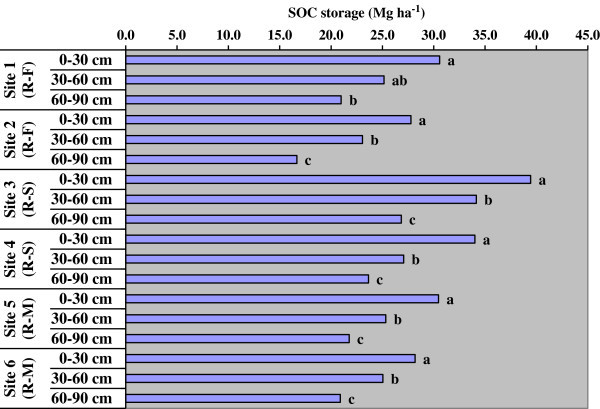
**Soil organic carbon (SOC) storage in different depths under different sites; R-F indicates rice-fallow, R-S rice-sugarcane rotation and R-M is rice-mung bean cropping; vertical bars with same letter are not significant at p < 0.05 at a site as per DMRT.**

Depth-wise soil organic carbon (SOC) storage shows the highest in the first 30 cm soil depth (0-30 cm) and gradually decreased with depth increments significantly in each site (Figure 5). In the 0-30 cm soil layer, SOC storage ranged from 27.77 Mg ha^-1^ under rice-fallow system in site 2 to 39.44 Mg ha^-1^ under rice-sugarcane in site 3. In the 30-60 cm soil layer, SOC storage ranged from 23.06 Mg ha^-1^ under rice-fallow system in site 2 to 34.15 Mg ha^-1^ under rice-sugarcane in site 3. In the 60-90 cm soil layer, it varied from 16.67 Mg ha^-1^ under rice-fallow system in site 2 to 26.83 Mg ha^-1^ under rice-sugarcane in site 3. The SOC storage significantly decreased towards greater depth of soils as evident from estimated values.

### Development of soil pedotransfer functions under different cropping

**Figure 6:**
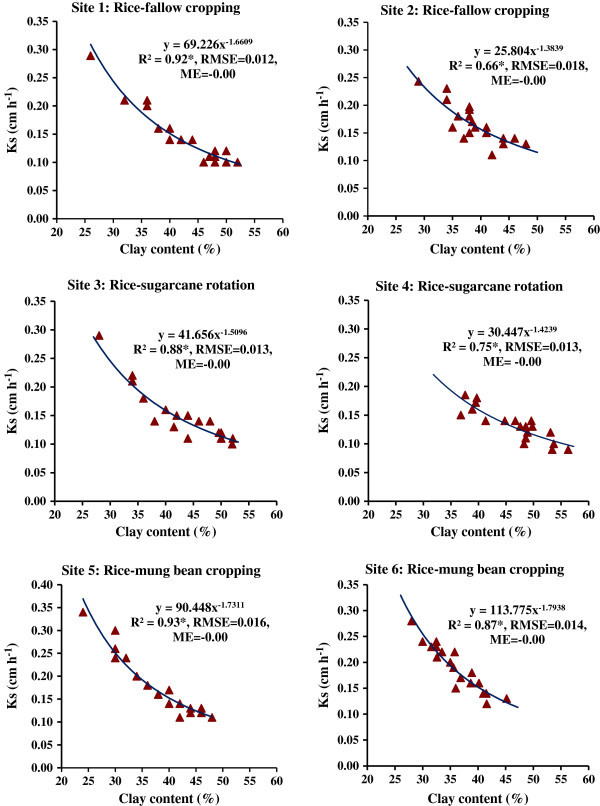
**Pedotransfer functions (PTFs) for prediction of saturated hydraulic conductivity (Ks) using clay content of soils from different sites under major cropping systems in the command area; R** ^**2**^ , coefficient of determination (*significant at p < 0.05); RMSE, root mean square error; ME, mean error.

Pedotransfer functions (PTFs) were developed for saturated hydraulic conductivity (Ks) and water retention at FC (-33 kPa) and PWP (-1500 kPa) using the data set of textural values viz. clay (%), sand fractions (%) and clay + silt (%). Prediction results revealed that the clay (%) was much better predictor variable than sand (%) or clay + silt (%) for prediction of Ks, FC and PWP. The PTFs for Ks were best represented by the power functions with considerably higher and significant values of R^2^ at P < 0.05 for every site and cropping system (Figure 6).

**Figure 7:**
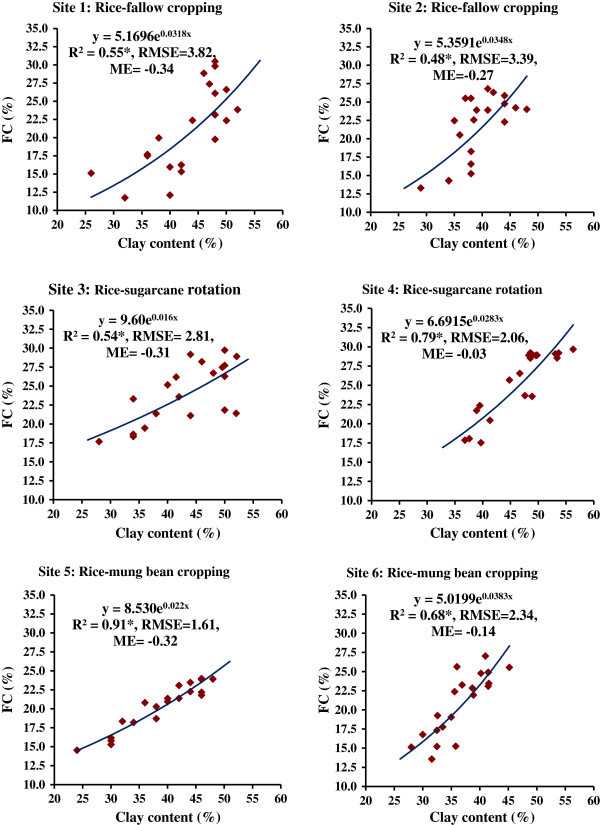
**Pedotransfer functions (PTFs) for water retention at field capacity (FC, -33 kPa) using clay content of soils from different sites under major cropping systems in the command area; R** ^**2**^ , coefficient of determination (*significant at p < 0.05); RMSE, root mean square error; ME, mean error.

**Figure 8:**
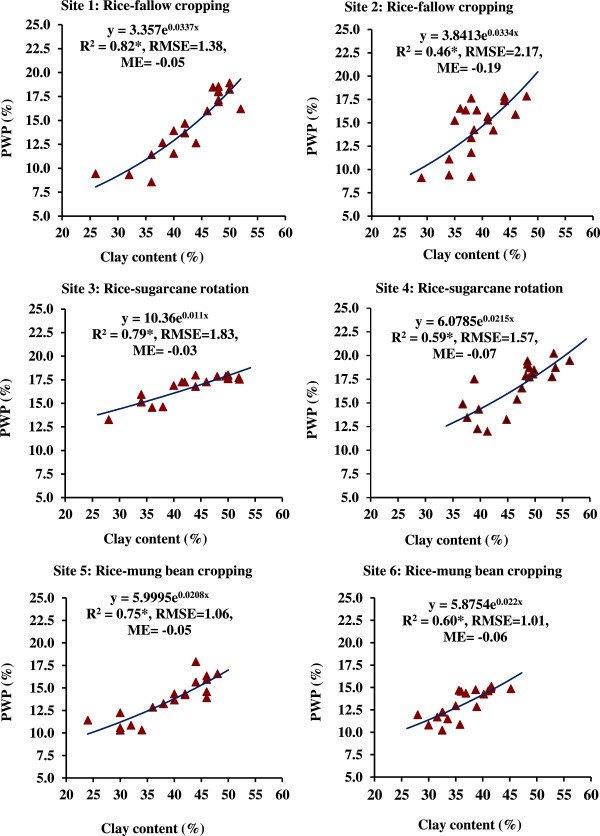
**Pedotransfer functions (PTFs) for water retention at permanent wilting point (PWP, -1500 kPa) using clay content of soils from different sites under major cropping systems in the command area; R** ^**2**^ , coefficient of determination (*significant at p < 0.05); RMSE, root mean square error; ME, mean error.

The RMSE for six PTFs were also low indicating a stronger relationship with greater accuracy. A significantly (P < 0.01) strong and negative correlation was obtained between Ks and clay (%) as indicated by the correlation coefficient (r) of -0.93^**^ for site 1, -0.78^**^ for site 2, -0.91^**^ for site 3, -0.83^**^ for site 4, -0.95^**^ for site 5 and -0.94^**^ for site 6. The PTFs for water retention at FC and PWP (Figures 7 and 8) were found to be exponential functions using clay content (%) as the predictor variable with significant R^2^ (P < 0.05) for every study site. A strong positive and significant (P < 0.01) correlation was found between clay content and FC or PWP for every site as evident from r values ranging from 0.68^*^ (P < 0.05) to 0.89^**^ (P < 0.01) and from 0.71^*^ (P < 0.05) to 0.90^**^ (P < 0.01) for FC and PWP, respectively.

## Discussion

An important finding was that the average clay content especially in the lower soil layers under rice-sugarcane systems in sites 3 and 4 was comparatively greater than other sites. Sand content decreased towards lower soil layers in every cropping system; whereas clay contents increased gradually towards lower soil depths. Because of high clay content, those exceeded 40%, the soils of lower layers are mostly clayey. Due to heavy rainfall during rainy season and tillage operations for rice cultivation, the clay fractions were greater in the lower layers. This might be due to the clay migration from upper soil to the lower layers. The difference in settling of soil particles in the soil layers due to repeated plouging might have attributed to the difference in sand and clay contents in different sites. Agoumé and Birang (2009) also reported similar results for the fields of Ngoungoumou village near Ebolowa in Cameroon. Clay accumulation in the sub-soil could result in reduced porosity, increased water retention and reduced drainage (Agoumé and Birang 2009). Site specificity and existing cropping systems affected the sand, silt and clay fraction of soils of our study area. Silt contents in these soils were low; however there was no definitive trend of increase or decrease in silt content due to the depth variable. Voundi Nkana and Tonye (2003) also did not find any effect of cropping systems on the silt fraction distribution having a very less silt content.

Comparatively lower bulk density values were obtained in the sites 3, 4 and 5 under rice-sugarcane and rice-mung bean cropping systems. This difference was not easily explainable but might be ascribed to the compaction of the topsoil due to repeated ploughing for cropping practices (Agoumé and Birang 2009). In general, higher BD might be ascribed to the higher clay content and compaction due to repeated ploughing and cropping practices for a long time. The significant decrease in saturated hydraulic conductivity values towards greater depths of soil profile for each site under different cropping systems indicated the greater clay contents in lower depths of soils. Among all sites, Ks values were lower in the sites 3 and 4 having higher clay content in rice-sugarcane crop rotation. The difference and the trends are in conformity with the sand and clay contents. The results are in agreement with the research findings of McBratney et al. (2002), Santra and Das (2008) and Wösten et al. (2001). Of course, the aggregate size also determines the water transport in soils (Sławiñski et al. 2011). Water retention at field capacity and permanent wilting point increased significantly with the increase in soil depth for every site. The reasons and underlying factors for difference in PWP values were mostly governed by the difference in clay contents. Available water capacity was greater at sites 3, 4 in the rice-sugarcane system. Basically, it was the clay fractions in the soils of different sites which determined the higher FC, and in turn the higher AWC of soils. The available water capacity was also related to the soil organic matter content of soils as was evident from higher SOC values. Our results match with the finding of Gol (2009).

The greater organic carbon content (SOC) in rice-sugarcane was due to the continuous cropping with higher rates of fertilizer, year round cropping practice for sugarcane that might have sequestered greater SOC in sites 3 and 4. Moreover, sugarcane crop residues viz. trashes and greater root biomass from a long duration crop might have positively contributed to the higher organic carbon content in these soils. The extensive root systems i.e., formation of new roots and decay of old roots added the considerable amount of organic matter to the soil. The comparatively greater SOC in the soils of rice-mung bean system might be ascribed to the greater soil organic matter and lower loss of carbon through release of CO_2_ into the atmosphere. Similar results were reported by previous researchers, that the SOC content in croplands was strongly correlated with crop and soil management practices (Hao et al. 2002; Mandal et al. 2012). The SOC storage significantly decreased towards greater depth. This was plausibly due to the greater organic carbon content in sites 3 and 4 under rice-sugarcane crop rotation systems. The variation in total SOC storage in different site was primarily due to differences in soil organic carbon content and bulk density of soil. Similar results were found by previous researchers (Shibu et al. 2010).

Clay content was positively and significantly correlated with BD, FC and PWP, and negatively correlated with Ks. Silt was not very well correlated with other parameters. This trend was mostly similar in every study site. It revealed that organic carbon content, bulk density and clay contents appeared to be the important soil properties to improve estimation of soil water retention from soil texture. The higher SOC in the soils of sites 3 and 4 led to greater water retention and lesser Ks. This might be related to the fact that the structure-forming effect of organic matter positively affected the water retention at FC and PWP. The water retention of organic matter itself is a probable reason of the effect of organic carbon on water retention at -1500 kPa although the organic matter is known to modify the availability of adsorption sites of clay minerals to water. Clay is inversely related to Ks. It was observed that clay content (%) rather than clay + silt content (%) was better predictor with power functions for the response variable Ks. Of course, the nature of power functions for different sites were different. The values of coefficient of determination (R^2^) were significant at 5% level, which indicated the significant contribution of the function with clay content as predictor variable to the variation in Ks, FC and PWP. Mean error (ME) values were negative indicating underestimation of response variables viz. Ks, FC (-33 kPa) and PWP (-1500 kPa); but the degree of ME were very close to zero which indicates that there were least bias of the estimate for each prediction function. Hence, the uncertainty of developed PTFs was very less for prediction of Ks, FC and PWP in the area of our study; clay content values may be used for precise prediction of saturated hydraulic conductivity. The PTFs may be utilized for different models. The FC and PWP could be predicted well with clay content (%) as predictor variable.

## Conclusions

This study characterized the soil properties like particle size fractions, bulk density, saturated hydraulic conductivity, soil organic carbon and also pH for the cropping area under Kuanria command in Odisha, an eastern Indian state. A higher clay fraction in soils of rice-sugarcane crop rotations indicates greater water retention and lower hydraulic conductivity than rice-mung bean and rice-fallow systems.

It is concluded that the sites of rice-sugarcane rotation had greater soil water retention at FC and PWP, due to higher clay content in the soils. This has the implication on better management of rainwater as well as the irrigation water which is supplied from reservoir through canal systems. There will be better water holding capacity in these soils, and less loss of water due to deep percolation. As the saturated hydraulic conductivity in the sites of rice-sugarcane was less, the construction of water storage structures will be successful for multiple purposes like fish culture and providing irrigation to crops during dry spells. Since bulk density was slightly higher, it is concluded that the soils are mostly compact especially in the lower layers, and have less organic matter. Soils of rice-sugarcane rotation showed comparatively low bulk density due to greater organic matter. Soil organic carbon (SOC) status was low (0.34-0.95%); soils with rice-sugarcane rotation system had greater organic carbon storage than other rice-based systems. The SOC storage information would help for future crop planning concerning soil organic carbon restoration and other soil properties especially for this command.

In our study, pedotransfer functions were power functions for estimation of saturated hydraulic conductivity, and the exponential functions for water retention at FC and PWP using clay content. The site-specific PTFs for soils under rice-fallow, rice-sugarcane and rice-mung bean systems would be useful to obtain the values of hydraulic conductivity and water retention, and their use as model parameter for studies on water movement in the profile, solute transport and environmental problems etc. Even, these PTFs may be utilized for soils of elsewhere having similar situations, rainfall pattern, basic soil characteristics and cropping practices.
